# Consequences of Epistasis on Growth in an Erhualian × White Duroc Pig Cross

**DOI:** 10.1371/journal.pone.0162045

**Published:** 2017-01-06

**Authors:** Lucy Crooks, Yuanmei Guo

**Affiliations:** 1 State Key Laboratory for Pig Genetic Improvement and Production Technology, Jiangxi Agricultural University, Nanchang, China; 2 Department of Animal Breeding and Genetics, Swedish University of Agricultural Sciences, Uppsala, Sweden; 3 Sheffield Diagnostic Genetics Service, Sheffield Children's NHS Foundation Trust, Sheffield, United Kingdom; College of Agricultural Sciences, UNITED STATES

## Abstract

Epistasis describes an interaction between the effects of loci. We included epistasis in quantitative trait locus (QTL) mapping of growth at a series of ages in a cross of a Chinese pig breed, Erhualian, with a commercial line, White Duroc. Erhualian pigs have much lower growth rates than White Duroc. We improved a method for genomewide testing of epistasis and present a clear analysis workflow. We also suggest a new approach for interpreting epistasis results where significant additive and dominance effects of a locus in specific backgrounds are determined. In total, seventeen QTL were found and eleven showed epistasis. Loci on chromosomes 2, 3, 4 and 7 were highlighted as affecting growth at more than one age or forming an interaction network. Epistasis resulted in both the QTL on chromosomes 3 and 7 having effects in opposite directions. We believe it is the first time for the chromosome 7 locus that an allele from a Chinese breed has been found to decrease growth. The consequences of epistasis were diverse. Results were impacted by using growth rather than body weight as the phenotype and by correcting for an effect of mother. Epistasis made a considerable contribution to growth in this population and modelling epistasis was important for accurately determining QTL effects.

## Introduction

Epistasis describes the effect of a genetic locus changing with the genotype at another locus. In quantitative genetics, it is represented as an interaction between the effects of loci. Epistasis is generally not considered in QTL mapping although it may significantly contribute to phenotypic variation [1,2]. Evaluating epistasis can help identify genetic networks and provide a greater understanding of the biology underlying complex traits. It should increase the accuracy of estimated effects. Accurate estimates are fundamental to designing successful breeding schemes using the QTL. It is particularly important to know if the effect of a locus alters direction depending on the genetic background.

Erhualian is a Chinese indigenous pig breed. In the past, pig breeding in China was mainly local, which has resulted in geographically distinct breeds. White Duroc is a cross of the Duroc breed, which originated in the United States but is believed to have at least partial European ancestry, with a European white-coloured breed. Erhualian pigs produce large litters and have good offspring survival rates but a slow growth rate. White Durocs have higher growth rates and less body fat. Chinese and European pigs appear to be the result of separate domestications of local wild boar populations around 8,000–6,000 years ago [3,4]. Sequencing data suggests that Chinese and European wild boars diverged around 1 million years ago [5]. European and Chinese pigs also cluster to separate groups based on microsatellite data, with Durocs at the base of the European cluster and Erhualian and other breeds from the same area, furthest from the European cluster [6].

Pigs have sigmoidal growth curves that are typical of mammals, where their growth rate initially increases with age and then declines as individuals reach their mature body size [7]. We examined growth at a series of ages. The allocation of resources to various tissues is likely to shift with time, therefore distinct QTL are likely to be identified at different ages. In contrast, QTL that are most fundamental for growth should be detected at multiple ages. Investigating epistasis will provide a fuller picture of the genetic factors affecting growth and may identify new loci or effects of known loci at further ages. Additionally, QTL that interact with more than one locus can have an increased impact because of their influence on other genes.

There are few assessments of epistasis in pigs. In the population we analyse here, two pairs of epistatic QTL for body dimensions and organ weights were identified [8]. A pair of QTL with joint effects on late body weight in an Iberian with Landrace cross was found [9] but the interaction between these loci was not significant. However, several studies have explored QTL for pig growth at different ages without investigating epistasis. Ai et al. [10] previously analysed body weight in this population. They found loci on chromosomes 4, 7 and 8 with effects at more than one age but these findings may have been generated by correlations between body weights over time. Growth QTL in similar positions on chromosomes 4 and 7 were detected at more than one age in a cross between Meishan, a breed from near to the Erhualian, and European Large Whites [11]. A combined analysis of seven populations generated from Meishan or European wild boar crossed with Western commercial breeds also found a QTL in the same region of chromosome 4 that influenced birth weight and overall growth [12]. Two studies of Meishan and White crosses detected QTL in similar positions on chromosome 1 effecting early growth and weight at two ages [11,13]. A QTL near that location also had chromosome-wide significant effects on growth over two weight intervals in a cross between Western commercial lines [14].

Studies in chicken have shown epistasis can be important for growth. In a cross between White Leghorns and a Red Junglefowl, 11 of 17 QTL affecting hatch weight or growth were involved in epistasis [15]. The additional QTL identified by testing for epistasis explained up to 10% of the residual trait variance. With chickens from a divergent selection experiment, assessing the individual effects of loci only identified one QTL for weight at the selection age [16]. Epistatic analysis showed this locus was the centre of a network of five other QTL, which together explained 45% of the weight difference between the parental lines at the selection age [17]. Interestingly, when growth was investigated rather than weight, there were no more than minimal networks of three loci at each age [18].

When epistasis is detected, we want to know what the impact is on the phenotype. Epistatic models are usually formulated with interaction variables that are products of additive and dominance variables for each locus and epistasis has been described by which interaction terms are significant, for example as additive-by-additive or additive-by-dominance [19]. However this does not make the effects of each locus explicit. It is also hard to infer the phenotypic consequences if more than one interaction term is significant. Here, we propose a new approach for representing epistasis. We estimate separate additive and dominance effects of a locus for each genotype at the other locus and report the significant effects. We provide a mathematical framework for calculating the effects and their standard errors.

We mapped QTL by their independent and epistatic effects on growth at different ages in an Erhualian and White Duroc F_2_ cross. We were particularly looking for loci with effects at several ages or that interacted with more than one QTL. To reduce the impact of correlations between body weights over time, we examined weight increase, corrected for effects of start weight. We also adjusted for an effect of mother and other explanatory variables. We refined a method for testing epistasis, primarily in how to correct for the individual effects of loci and by generating heat maps to guide the process. Our new approach was applied to the phenotypic outcome of epistasis. The effects of loci at varied ages and in different interactions were compared. To show the importance of considering epistasis in QTL mapping, we measured the variance explained and effect sizes of epistatic and independent QTL, determined how many epistatic loci were detected by a standard QTL scan, and looked at the change in estimated effects when epistasis was omitted.

## Materials and Methods

### Animals

The F_2_ cross has been described previously [20] and QTL for multiple phenotypes have been identified in these animals. The population was generated from 17 Erhualian sows and 2 White Duroc boars. The F_1_ generation comprised 59 sows with 9 boars and 1912 F_2_ individuals were produced in six batches and four parities. A simulation study found that for a population of 840 F_2_ individuals, the power to detect epistasis when the interaction of the QTL accounts for 2.5% of the total phenotypic variance, was about 7–35% [21]. Therefore, a population approximately double this size (when taking into account normal mortality) was chosen to maximise the opportunity to detect epistasis. Some pigs were fostered and pigs were kept with their mother or foster mother for 46 days. At this time the mother was removed. At around 80 days, pigs were separated into male and female groups of 6–10 individuals. Individuals from the same family were generally kept together but large families were split up and small families combined. Three-quarters of the males were castrated at a range of ages. More than half the pigs were moved to a different location, also at a range of ages. Nearly three-quarters of individuals were starved for 24 hours before the last weight measurement.

Serious consideration was given to pig welfare and the guidelines for the care and use of experimental animals established by the Ministry of Agriculture of China were adhered to. The ethics committee of Jiangxi Agricultural University approved this study. A veterinarian inspected the pigs twice a day looking for signs of illness including diarrhoea, cough, fever, lameness, impaired growth, hernia and rectal prolapse. Pigs were also tested by ELISA at days 18, 46 and 120 for antibodies to five infectious diseases including swine fever, Brucellosis and PRRS, which were prevalent in the area. Pigs were euthanized if they remained ill for more than three days, showed the abnormalities described above, or had above threshold levels of specific antibodies in accordance with the disease action plan determined by the veterinarian. Euthanasia was carried out by electric shock before bleeding according to Chinese industry standards (GBT 17236–1998).

Pigs were kept in solid floor pens inside several barns. Each pregnant sow was housed in an 8 m^2^ sty with a roof, window and door. The sty had a 1.8 m^2^ area only accessible to piglets to provide some protection from the mother, and a connected 6 m^2^ playground with no roof. After weaning, pigs were fed *ad libitum* on a diet containing 21% crude protein, 3300 kJ digestible energy and 1.25% lysine, and water was available *ad libitum*. Larger pigs were moved to 24 m^2^ sties and their diet was changed to 16% crude protein, 3100 kJ digestible energy and 0.78% lysine with water available *ad libitum*. Outside breeding, sows were kept in groups of 4–6 in a 12 m^2^ sty with a 6 m^2^ playground. Breeding boars were kept individually in 6 m^2^ sties with a 6 m^2^ playground. Young boars that were being kept for breeding were similarly housed but two per pen. Manure was cleared form the pens twice a day.

There were 226 piglets that died before the experimental endpoint. This is 12%, which is below the 16% mortality rate from live birth to slaughter, found nationwide for pig farms in the United States in 2012 [22]. The deaths correspond to an average of 1.5 piglets per litter. This might be expected, given that there is often a runt in a litter and these have higher mortality rates. Crosses between distinct breeds tend to have a higher number of runts. There were 134 piglets euthanised because of illness. A further 51 were crushed by a sow, this is a known problem in pig rearing and happens when the mother changes position; it is higher when mothers are not constrained, as in this study. Sows also killed 5 piglets by biting them. There were 14 piglets that died of hypothermia. Again, this is a known cause of piglet mortality; piglets have difficulty in regulating their body temperature, in part because they lack brown adipose tissue. Another 13 piglets died unexpectedly. Postmortem examinations were conducted by the veterinarian but the cause of death is unknown. The remaining 9 piglets died due to complications following blood sampling. At the end of the experiment, the majority of pigs were killed by electric shock before bleeding according to Chinese industry standards (GBT 17236–1998). Some pigs were kept to produce an F_3_ generation.

### Genotyping

Genotyping was carried out on all F_0_ and F_1_ individuals and 1757 F_2_ as previously described [23]. Data from 178 microsatellites covering the 18 autosomes were used in this study. Male and female specific linkage maps were created using CRI-MAP [24]. Sex specific marker positions were converted from Kosambi to Haldane map distances for interval mapping because the method assumes no interference in recombination. The lengths of the male and female autosomal maps were 2,361 and 3,215 cM, respectively. The number of markers per chromosome ranged from 5 to 24 and the mean sex-averaged distance between markers was 17 cM.

### Phenotyping

QTL mapping was performed on the F_2_ generation. Animals were weighed at birth, 21 and 46 days old and approximately 120, 210 and 240 days old. Traits analysed were birth weight and change in weight between the following ages: birth to 21 days (G_0-21_), 21–46 days (G_21-46_), 46–120 days (G_46-120_), 120–210 days (G_120-210_), 46–210 days (G_46-210_) and 210–240 days (G_210-240_). The combined interval of 46–210 days was analysed as well as 46–120 and 120–210 days because fewer individuals were measured at 120 days than the other time points.

### Data preparation

There was variation in the age when the later weight measurements were taken. To reduce this variation, only animals measured within the following ages were included: G_46-120_, 119–130 days for the second measurement; G_120-210_, 119–130 days for the first measurement and 209–213 days for the second measurement, with an interval of 80–90 days; G_46-210_, 205–213 days for the second measurement; G_210-240_, 207–211 days for the first measurement and 237–242 days for the second measurement, with an interval of 27–34 days. Individuals whose weights declined over the course of the experiment, except for the final weight after starvation, or that died before the end of the experiment were excluded from the analysis. Females from batches 1 and 2 were excluded from the analyses of growth after 46 days because they were fed a different diet.

The residuals after fitting explanatory variables to the phenotypes were used for the QTL analysis. A set of variables was tested for each age and only those that were significant at *p* < 0.05 were included. Correcting for other variables should increase the power to detect QTL by reducing the residual variance. Weight at the start of an interval was tested because we wanted to identify QTL that affect growth independent of start weight. Effects of sex, batch, mother and parity were evaluated for all ages. For fostered pigs, the foster mother and her parity were used for growth after birth. Number of offspring and number of live offspring were considered as covariates for birth weight and group size at the first measurement age for intervals starting before 120 days. For intervals where weight was not recorded at a fixed age, effects of measurement age were tested, as well as measurement interval for G_120-210_ and G_210-240_. Effects of age when moved and castration were checked for growth after day 46. Age when moved was first treated as a factor with levels spanning six-day intervals, then simplified by grouping together consecutive levels with similar effect estimates and finally checking different boundaries between levels to maximise the explained sum of squares. An effect of starvation was tested for G_210-240_.

Variables were fitted together in a mixed model with mother as a random effect and the other variables as fixed effects. Modelling was performed in R version 2.15.1 [25]. A restricted maximum likelihood (REML) method in the lme4 package version 0.999375–28 was used. Fixed effects were tested by *F* tests with the estimated residual variance as the denominator. In mixed models, the *F* distribution may not be a good approximation of the distribution of this test statistic and the appropriate denominator degrees of freedom is unclear. We estimated *p* using a *χ*^2^ approximation for *d × F*, where *d* is the degrees of freedom for the effect, under the assumption that the denominator degrees of freedom is large. The effect of mother was tested by a likelihood ratio test of the REML likelihoods for models with and without mother. Non-significant effects were sequentially removed in order of highest *p* value. The model was refit after each removal and the significance tests were recalculated. The model of significant explanatory variables is referred to as the base model. The phenotypes and explanatory variables in the base models are given as supplementary information in S3–S9 Texts.

### Data analysis

#### Independent QTL analysis

Firstly, loci were assessed assuming that they acted independently, i.e. without considering epistasis. The method from Haley and Knott [26] was used but with the probabilities of the alleles at each position originating from the Erhualian (*E*) or White Duroc line (*W*) calculated by triM [26,27]. triM uses information from all markers on a chromosome as proposed by Haley *et al*. [28] but applies a hidden Markov model algorithm, which can handle many more partially informative markers. Additive (*A*) and dominance (*D*) variables at 1 cM intervals were calculated as *A* = *p*(*WW*)–*p*(*EE*) and *D* = *p*(*EW*), where *p*(*X*) is the probability of having genotype *X*. The *A* and *D* values for each cM position are given in S2 Text. The following linear model was fit for each position:

$$\tilde{y}=\mu +aA+dD+\varepsilon$$ (1)

where $\tilde{y}$ is the adjusted phenotype, *a* and *d* are the additive and dominance coefficients, respectively, for the tested position and *ε* is a normally distributed error. This model and the following epistatic models are in the *F*_∞_ formulation [29]. The mean value of the phenotype for individuals with a given genotype is called the genotypic value, and *μ* is the mean of the genotypic values for the two homozygotes. The additive coefficient is half the difference between the genotypic values of the homozygotes. The dominance coefficient is the difference between the genotypic value of the heterozygote and the mean of the homozygote genotypic values.

*F* ratios were calculated for the combined additive and dominance coefficients. A genomewide significance threshold of *p* < 0.05 was determined for each trait by 1000 permutations of the phenotypes relative to the genotypes [30]. For each chromosome where the *F* ratio exceeded the threshold, the position with the highest *F* ratio was called a putative QTL. We also recorded the set of surrounding positions with values above the significance threshold, which we call the QTL span. If there was more than one set of positions above the threshold separated by only a small gap, the total stretch from the lowest to highest position was taken as the QTL span. The additive and dominance variables for all putative QTL were then added to model (1) and the model fit again to search for additional QTL using the same significance threshold. The process was repeated until no more putative QTL were found.

#### Epistatic QTL analysis

Secondly, pairwise epistasis was evaluated. We used a similar approach to Carlborg et al. [31] but with some amendments. The following linear model was fit to all pairs of positions:

$$\tilde{y}=\mu +a_{1}^{\prime }A_{1}+d_{1}^{\prime }D_{1}+a_{2}^{\prime }A_{2}+d_{2}^{\prime }D_{2}+i_{aa}I_{AA}+i_{ad}I_{AD}+i_{da}I_{DA}+i_{dd}I_{DD}+\varepsilon$$ (2)

where *A*_1_, *D*_1_, *A*_2_ and *D*_2_ are the additive and dominance variables for the first and second positions tested, respectively, and $a_{1}^{\prime },\;d_{1}^{\prime },\;a_{2}^{\prime },\;\text{and}\;d_{2}^{\prime }$ are additive and dominance coefficients for the first and second position, respectively. The additive and dominance coefficients are represented with a *'* because they do not have the same interpretation as in the single locus model ([29] and see Epistatic effects). The *I* are interaction variables generated by multiplying together the additive or dominance variable for the first position, as indicated by the first letter in the subscript, with the additive or dominance variable for the second position, as indicated by the second letter in the subscript, for example *I*_*AD*_ = *A*_1_*D*_2_. The parameters *i*_*aa*_, *i*_*ad*_, *i*_*da*_ and *i*_*dd*_ are additive by additive, additive (at the first locus) by dominance (at the second locus), dominance (at the first locus) by additive (at the second locus), and dominance by dominance coefficients, respectively. *μ* is the mean genotypic value for genotypes that are homozygotes at both loci.

The residual sum of squares (SSE) was used for significance testing, with a lower SSE corresponding to a better fit of the model to the data. A genomewide SSE threshold of *p* < 0.05 (SSE_genome_) was determined by 1000 permutations of the phenotypes relative to the genotypes using a genetic algorithm [32]. Only pairs of positions with a SSE lower than SSE_genome_ were kept.

The SSE is lowered for pairs involving a position close to a putative QTL because of the effect of that locus, and this needs to be corrected for. It was suggested that when one locus in a pair was within 10 cM of an already detected QTL a modified significance test should be used [33]. We instead propose that the modified test should be applied to all positions within the QTL span found in the independent QTL analysis; hence the breadth of positions is tailored to each locus. To visualise the impact of individual loci and guide the testing process, we plotted SSE_genome_-SSE as a heat map for the retained pairs.

Locus-specific SSE thresholds of *p* < 0.05 (SSE_specific_) were determined for each putative QTL from the independent QTL analysis by permutation [33]. An epistatic model of the putative QTL was fit with every other genome position as the interacting locus and the A and D variables of the other position and the interaction variables permuted; 1000 permutations were used. All pairs involving a position within the QTL span were compared against SSE_specific_ and only pairs with an SSE below SSE_specific_ were kept. When both positions in a pair were inside a QTL span, the pair was tested against the lowest SSE_specific_.

A heat map of SSE_specific_-SSE, where appropriate, and SSE_genome_-SSE otherwise, was generated for the remaining pairs. These maps showed small clusters of possible epistatic pairs. For each cluster, the pair with the highest value in the heat map (lowest SSE beneath the respective threshold) was found. Because there is a hard boundary (the edge of the QTL span) where SSE_specific_ is used, parts of regions in the initial heat map can remain adjacent to the boundary whilst pairs inside the boundary are removed. Comparing the heat maps shows such edge effects. When we observed this, we checked if there was a pair with a lower absolute SSE inside the boundary and if so the cluster was ignored. Finally, the interaction of the loci in a pair was assessed by an *F* test of the four interaction coefficients together and *t* tests of each coefficient. Pairs with *p* < 0.05 for the *F* test or *p* < 0.0125 for any coefficient were called putative epistatic pairs.

#### Model simplification

The putative QTL and putative epistatic pairs were combined into a model of the raw phenotype with the explanatory variables to determine whether the loci were significant in this full model. When a locus in a putative epistatic pair matched with a putative QTL, which we defined as being within 20 cM or inside the region of the putative QTL, we assumed that they were the same locus and fit only the epistatic locus, using the position from the epistatic analysis. If there were similar positions amongst epistatic pairs, we included these as separate loci. The following mixed model was fit by REML:

$$y=\mu +X\beta +Z\gamma +\sum_{r}\left(a_{r}A_{r}+d_{r}D_{r}\right)+\sum_{s}(a_{s,1}^{\prime }A_{s,1}+d_{s,1}^{\prime }D_{s,1}+a_{s,2}^{\prime }A_{s,2}+d_{s,2}^{\prime }D_{s,2}+i_{s,aa}I_{s,AA}+i_{s,ad}I_{s,AD}+i_{s,da}I_{s,DD}+i_{s,dd}I_{s,DD})+\varepsilon$$ (3)

where *y* is the raw phenotype, *X* is the model matrix for fixed effects, *β* is the vector of fixed effect coefficients, *Z* is the model matrix for mothers and *γ* is the vector of random maternal effect coefficients. *a*_*r*_ and *A*_*r*_, for example, are the additive coefficient and additive variable for putative QTL *r*. *a*_*s*,1_, *i*_*s*,*ad*_ and *I*_*s*,*AD*_, for example, are the additive coefficient for locus 1 in putative epistatic pair *s* and the additive by dominance coefficient and variable for this pair. *μ* equates to the mean genotypic values of genotypes that are homozygotes at all loci after correcting for the effects of the explanatory variables.

Putative epistatic pairs were tested for a significant interaction in (3). The significance threshold was *p* < 0.05 in an *F* test of the set of interaction coefficients or *p* < 0.0125 in a *t* test of any individual interaction coefficient. Non-significant pairs were removed sequentially, in order of the highest *p* value for the set of interaction terms. If either locus matched a putative QTL, the locus was kept in the model as a putative QTL unless there was a similar position in a remaining putative epistatic pair. The position of the locus was reassessed if it was different for the removed pair and the putative QTL; the positions were compared in the current model and the one giving the highest log likelihood was used. The model was refitted after removing each pair and the significance tests were recalculated. When only significant pairs remained, any loci located within 20 cM of each other in different pairs were examined. We assumed that these were the same locus and replaced them with one locus in the model. The different positions were compared and the one with the highest log likelihood was taken. We report the loci remaining in pairs after this process as epistatic QTL.

We applied stringent thresholds for detecting epistasis, which means that true effects could be missed because of insufficient power. Given an expectation that some QTL will affect growth at more than one age, we tested whether the pairs of epistatic loci were significant at other ages. Pairs were tested by adding them separately to the current model, removing any matching putative QTL and evaluating the interaction as in the previous step. If more than one pair was significant, these pairs were also evaluated by adding them together to the current model, removing any matching putative QTL and testing the interaction as before. Pairs found significant were retained in the model. For distinction, we describe these loci as pointwise-significant epistatic QTL.

Finally, putative QTL in the model that were not in epistatic pairs were tested for significance in (3). The significance threshold was *p* < 0.05 in an *F* test of the additive and dominance coefficients combined or *p* < 0.025 in a *t* test of either coefficient. Loci satisfying this criterion are reported as independent QTL. In comparing QTL across ages, we considered loci within 30 cM of each other, whether independent or epistatic, as the same locus.

#### Variance explained by the QTL

The percentage residual variance explained by parts of the model was calculated as the increase in penalised residual sum of squares (PSS) of removing the relevant terms from the final model, divided by the PSS of the base model, and multiplied by 100. The contribution of pointwise-significant pairs was included for epistatic QTL.

#### Graphical representation of epistasis

We illustrate epistasis by plotting the genotypic values for each genotype combination. Genotypic values were calculated relative to the mean for homozygotes at all loci and assuming constant values for the explanatory variables. From (2), the genotypic values are given by

$$G_{WWWW}=a_{1}^{\prime }+a_{2}^{\prime }+i_{aa}$$

$$G_{EWWW}=d_{1}^{\prime }+a_{2}^{\prime }+i_{da}$$

$$G_{EEWW}={-a}_{1}^{\prime }+a_{2}^{\prime }-i_{aa}$$

$$G_{WWEW}=a_{1}^{\prime }+d_{2}^{\prime }+i_{ad}$$

$$G_{EWEW}=d_{1}^{\prime }+d_{2}^{\prime }+i_{dd}$$

$$G_{EEEW}={-a}_{1}^{\prime }+d_{2}^{\prime }-i_{ad}$$

$$G_{WWEE}=a_{1}^{\prime }-a_{2}^{\prime }-i_{aa}$$

$$G_{EWEE}=d_{1}^{\prime }-a_{2}^{\prime }-i_{da}$$

$$G_{EEEE}=-a_{1}^{\prime }-a_{2}^{\prime }-i_{aa}$$ (4)

where, for example, *G*_*EWWW*_ is the value for genotype *EW* at the first locus and *WW* at the second locus. To facilitate comparison between ages, genotypic values were standardised by dividing them by the square root of the residual variance from the base model. Different genotypes at the first locus are shown along the x-axis. Values for the same genotype at the second locus are joined by lines and different genotypes at the second locus are distinguished by the line type. The lines illustrate the change in phenotype with genotype at the first locus. Vertical distances between the lines at the positions of the x-axis tick marks show the change in phenotype with genotype at the second locus. Non-parallel lines are indicative of epistasis because the effects change with the genotype at the other locus.

For the QTL that interacted with two loci at the same age, the genotypic value depends on the genotypes at all three loci. The equations are given in S1 Text.

#### Epistatic effects

Additive and dominance effects for a locus were calculated separately for each genotype at the other locus. They were measured from the genotypic values analogously to the additive and dominance coefficients in the single locus model (1). Hence,

$$a_{1}(X)=\left(G_{WWX}-G_{EEX}\right)/2$$

$$d_{1}(X)=G_{EWX}-\left(G_{WWX}+G_{EEX}\right)/2$$

$$a_{2}(X)=\left(G_{XWW}-G_{XEE}\right)/2$$

$$d_{2}\left(X\right)=G_{XEW}-\left(G_{XWW}+G_{XEE}\right)/2$$ (5)

where *a*_*i*_(*X*) is the additive effect for locus *i* for genotype *X* at the other locus and *d*_*i*_(*X*) is the corresponding dominance effect. From (4) the set of effects are

$$a_{1}(WW)=a_{1}^{\prime }+i_{aa}$$

$$d_{1}(WW)=d_{1}^{\prime }+i_{da}$$

$$a_{1}(EW)=a_{1}^{\prime }+i_{ad}$$

$$d_{1}(EW)=d_{1}^{\prime }+i_{dd}$$

$$a_{1}(EE)=a_{1}^{\prime }-i_{aa}$$

$$d_{1}(EE)=d_{1}^{\prime }-i_{da}$$

$$a_{2}(WW)=a_{2}^{\prime }+i_{aa}$$

$$d_{2}(WW)=d_{2}^{\prime }+i_{ad}$$

$$a_{2}(EW)=a_{2}^{\prime }+i_{da}$$

$$d_{2}(EW)=d_{2}^{\prime }+i_{dd}$$

$$a_{2}(EE)=a_{2}^{\prime }-i_{aa}$$

$$d_{2}\left(EE\right)=d_{2}^{\prime }-i_{ad}$$ (6)

Provided the two loci are in linkage equilibrium, the average additive and dominance effects for a locus, weighted by the genotype frequencies at the other locus, are equivalent to the additive and dominance coefficients from the single locus model.

For the QTL that interacted with two loci at the same age, additive and dominance effects vary with the combination of genotypes at both other loci. The equations are given in S1 Text. Values for the other two loci only depend on the first locus and are calculated as in (6).

The significance of the additive and dominance effects was tested by *t* tests. The standard errors of the effects were calculated using the variances and covariances from the fitted model as shown in S1 Text. The *p* values for a pair were corrected for multiple testing by Holm’s method, which is a sequential Bonferroni approach and a significance threshold of *p* < 0.05 was used [34]. For the QTL that interacted with two loci at the same age, the set of effects for both pairs were tested together with a significance threshold of *p* < 0.1 after the Holm correction. The threshold was adjusted because we were assessing terms from two pairs, both of which had significant epistasis in the final model.

The additive and dominance effects of independent QTL are equal to the values of their additive and dominance coefficients. The significance of these effects was tested by *t* tests with a significance threshold of *p* < 0.025. To facilitate comparison between ages, epistatic additive and dominance effects and additive and dominance effects of independent QTL were standardised by dividing them by the square root of the residual variance from the base model.

#### Independent effects model

We also fit a model of the QTL that were identified by the independent QTL analysis without any interactions. Loci were fit to the raw phenotypes by REML. The model was

$$y=\mu +X\beta +Z\gamma +\sum_{t}\left(a_{t}A_{t}+d_{t}D_{t}\right)+\varepsilon$$ (7)

where *t* is the set of QTL identified by the independent QTL analysis. We refer to this as the independent effects model. The QTL were tested for significance at *p* < 0.05 in an *F* test of the additive and dominance coefficients together or *p* < 0.025 in a *t* test of either coefficient. The values for significant coefficients were standardised by dividing them by the square root of the residual variance from the base model. The additional percentage residual variance explained by the epistatic model over the independent effects model was calculated from the difference in PSS between the two models.

#### Conversion of QTL positions to the MARC map

QTL positions were converted to the MARC pig map [35] to facilitate comparison with loci in the pig QTL database [36]. The nearest markers flanking each QTL that were in both the study-specific and MARC maps were used and positions calculated by interpolation between these markers. The positions of the QTL on the original Haldane map are given in S1 Table.

## Results

The average growth rate increased up to day 210 and slightly dropped for days 210–240 (Table 1). Table 1 shows the number of animals included for each phenotype, the explanatory variables in each base model and the residual variance of the phenotypes after adjusting for the explanatory variables. There was a significant effect of mother at all ages. Sex was a significant factor for all the phenotypes except G_46-120_; males had heavier birth weights but females had higher weight gains. Growth increased with weight at the start of the interval except in the final period. Birth weight decreased with the total number of offspring and growth up to day 46 decreased with group size. After adjusting for the explanatory variables there was a low correlation between all the non-overlapping traits (maximum Pearson’s |*r*| = 0.13, Table 2). Several of these had been moderately correlated before the adjustment (Pearson’s *r* = 0.20–0.45). As expected, there was a moderate to high correlation between G_46-210_ and both G_46-120_ and G_120-210_ (Pearson’s *r* = 0.57 and 0.83, respectively).

**Table 1:** Description of traits and base models.

Trait	N^a^	Mean, kg	ADG^b^, kg/day	Explanatory variables	Residual variance
**BW**	1505	1.2	-	mother, sex, batch, n. offspring^c^, n. live offspring^d^	0.047
**G**_**0-21**_	1503	4.1	0.2	mother, sex, batch, weight, group size	0.71
**G**_**21-46**_	1501	6.0	0.24	mother, sex, batch, parity, weight, group size	2.4
**G**_**46-120**_	759	21	0.28	mother, batch, weight, age^e^	21
**G**_**120-210**_	684	48	0.53	mother, sex, batch, parity, weight, interval, castration	86
**G**_**46-210**_	1074	69	0.42	mother, sex, batch, weight, moved^f^, castration	140
**G**_**210-240**_	963	15	0.5	mother, sex, batch, age^g^, interval, moved^h^, castration, starvation	31

Mother was treated as a random effect and the other explanatory variables as fixed effects.

^a^Number of individuals.

^b^Average daily growth rate.

^c^Total number of offspring born.

^d^Number of offspring born alive.

^e^Age above 119 days for second weight measurement.

^f^Grouped into not moved, moved before day 105, and moved day 105 or after.

^g^Age above 207 days for first weight measurement.

^h^Grouped into not moved, moved before day 115, and moved day 115 or after.

**Table 2:** Pearson correlations between adjusted traits.

	G_0-21_	G_21-46_	G_46-120_	G_120-210_	G_46-210_	G_210-240_
**BW**	0.01	0.03	0.04	0.06	0.06	0.11
**G**_**0-21**_		-0.02	-0.03	-0.02	0.00	-0.02
**G**_**21-46**_			0.00	-0.13	-0.03	-0.02
**G**_**46-120**_				0.07	0.57	0.12
**G**_**120-210**_					0.83	0.04
**G**_**46-210**_						-0.01

**Fig 1:**
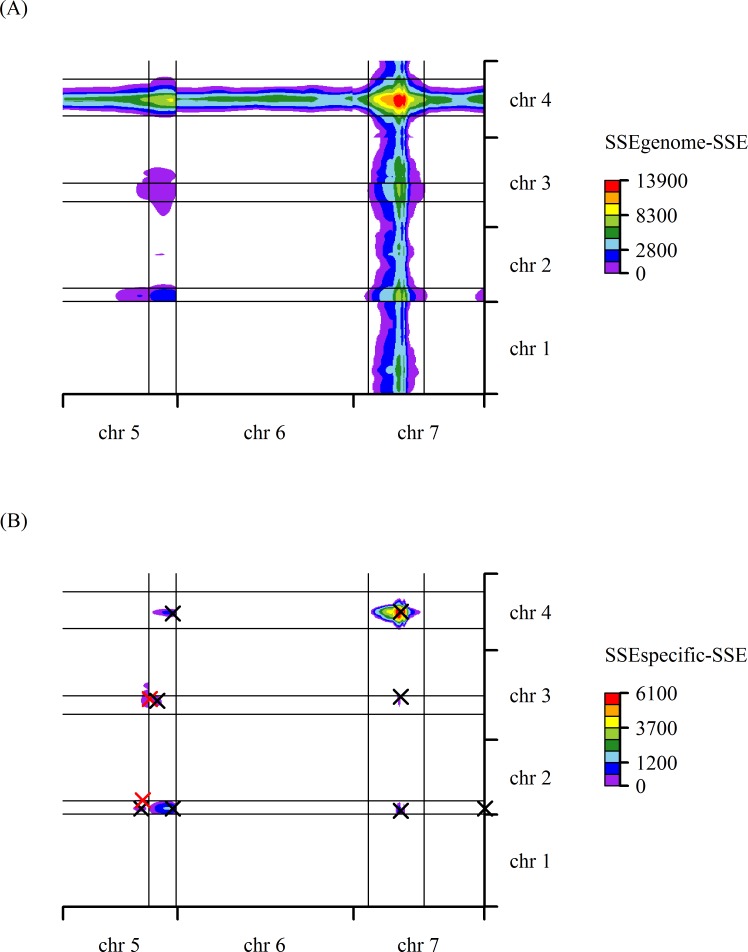
Process of epistasis significance testing. Graphic represents analysis of growth from 46–210 days for loci on chromosomes 1–4 paired with loci on chromosomes 5–7. The level of the residual sum of squares (SSE) below a significance threshold is shown for pairs of loci. Black lines represent QTL spans for loci identified in the independent QTL analysis. (A) Level of SSE below genomewide threshold (SSE_genome_). (B) Level of SSE below locus-specific threshold (SSE_specific_), where appropriate, and the genomewide threshold, otherwise. Black crosses show pairs taken forward for further testing and red crosses show pairs that were discarded because of edge effects.

Fig 1 illustrates the process we used to identify putative epistatic QTL for G_46-210_. Fig 1A shows the SSE for locus-pairs with a value below the genomewide significance threshold in a subset of the genome. The coloured region stretching horizontally across the graph reflects the effect of a locus on chromosome 4 and the coloured vertical slice, the effect of a locus on chromosome 7, both of which were identified in the independent QTL analysis. The black lines show the QTL spans determined from the independent analysis for these loci and loci on chromosomes 2, 3 and 5. A locus on chromosome 1 was also detected in the independent QTL analysis but no span for this locus is shown because the locus-specific significance threshold was higher than SSE_genome_ and therefore only the genomewide threshold was applied for this QTL region. Fig 1B shows the values of SSE_specific_-SSE within QTL spans and SSE_genome_-SSE elsewhere. Where two QTL spans crossed, the lowest SSE_specific_ was used. With the additional thresholds, only small clusters remain. The black crosses show the pair with the highest value in the heat map for each cluster. Red crosses show two instances where regions in Fig 1A were split into separate clusters because different thresholds were applied either side of the boundary of a QTL span. The pairs marked with the red crosses were discarded because a pair the other side of the boundary had a lower absolute SSE. Of the eight pairs indicated by black crosses in Fig 1B, four were significant when the interaction was tested, and only one, the interaction of a second locus on chromosome 7 with the chromosome 2 locus, remained after model simplification.

There were 17 QTL detected (Table 3), which together explained 1–27% of the residual phenotypic variance (Table 4). Eleven QTL showed epistasis in seven interacting pairs (Table 5). With pointwise significant pairs included, there was epistasis for five of the seven ages examined. There were between one and four epistatic pairs at these ages and together they explained 3–17% of the residual phenotypic variance (Table 4). No epistasis was seen for G_0-21_ and G_210-240_. There were between one and four independent QTL per age (Table 6) and together they explained 1–15% of the residual variance (Table 4). The highest numbers of both epistatic pairs and independent QTL were found for G_46-210_. Epistatic QTL explained more residual variance than independent QTL at three ages (Table 4). The relative contribution was highest for birth weight where the epistatic pair explained twice the residual variance of the independent locus.

**Table 3:** Growth QTL.

QTL	Chromosome	Position, cM	Trait	Epistatic
**Q1.1**	1	36	G_46-210_	
**Q1.2**	1	88	G_120-210_	Y*
		(88)	(G_46-210_)	y
**Q2.1**	2	(10)	(G_46-120_)	y
		10	G_46-210_	Y*
**Q2.2**	2	47	G_120-210_	Y
**Q3.1**	3	33	G_21-46_	Y
		29	G_46-210_	Y*
		42	G_120-210_	
**Q3.2**	3	88	G_21-46_	
**Q4.1**	4	72	G_21-46_	
		71	G_46-120_	
		66	G_120-210_	Y*
		64	G_46-210_	
**Q4.2**	4	128	BW	Y
		(128)	(G_46-120_)	y
**Q5**	5	112	G_46-210_	
**Q6.1**	6	(17)	(G_120-210_)	y
		17	G_46-210_	Y
**Q6.2**	6	88	G_21-46_	
		98	G_46-120_	
**Q7.1**	7	59	BW	Y*
		64	G_21-46_	Y*
		59	G_46-120_	y
		64	G_120-210_	
		58	G_46-210_	
		64	G_210-240_	
**Q7.2**	7	(156)	(G_46-120_)	y
		156	G_46-210_	Y
**Q8**	8	38	G_120-210_	Y
		38	G_46-210_	y
**Q9**	9	79	G_0-21_	
**Q13**	13	(58)	(G_120-210_)	y
		58	G_46-210_	Y
**Q18**	18	17	BW	

QTL are shown by chromosome with the traits for which they were found and the position identified for each trait, calibrated to the consensus pig linkage map. Epistatic effects are indicated by Y, with * showing QTL that were also detected by the independent QTL analysis. Pointwise-significant epistatic QTL are indicated by y and shown in brackets if they were not detected by the independent QTL analysis.

**Table 4:** Percentage residual variance explained by QTL.

	BW	G_0-21_	G_21-46_	G_46-120_	G_120-210_	G_46-210_	G_210-240_
**Independent**	1.5	1.2	4.9	6.6	9.1	14.8	2.0
**Epistatic**	3.0	-	2.6	8.0	17.3	10.4	-
**All QTL**	4.4	1.2	8.4	14.4	26.8	27.3	2.0

The variance explained was calculated by removing the terms from the full model. Therefore, if there are correlations between the independent and epistatic variables, the sum of the variances explained by each will be lower than the total variance explained.

**Table 5:** Epistatic pairs.

Trait	Pair(s)	% Var explained^a^	% Epistatic^b^
BW	Q7.1 x Q4.2	3.0	1.9
G_21-46_	Q7.1 x Q3.1	2.6	1.5
G_46-120_	(Q7.1 x Q4.2)	5.4	1.5
G_46-120_	(Q2.1 x Q7.2)	2.2	1.4
G_120-210_	Q1.2 x Q8	6.8	2.8
G_120-210_	Q2.2 x Q4.1	6.6	1.8
G_120-210_	(Q13 x Q6.1)	2.2	1.5
G_46-210_	Q13 x Q6.1	2.1	1.9
G_46-210_	Q2.1 x Q7.2 and Q2.1 x Q3.1	5.7	1.2, 0.7^c^
G_46-210_	(Q1.2 x Q8)	1.6	0.7

Pointwise-significant pairs are shown in brackets.

^a^Percentage residual variance explained by QTL together.

^b^Percentage residual variance explained by the interaction terms of pairs.

^c^First value is for Q2.1 x Q7.2, second value is for Q2.1 x Q3.1.

**Table 6:** Independent QTL.

Trait	QTL	% Variance explained^i^	Effects^ii^(s.e.)	
			a	d
BW	Q18	1.5	0.17(0.05)	0.23(0.08)
G_0-21_	Q9	1.2	-0.13(0.04)	-0.19(0.06)
G_21-46_	Q4.1	2.5	0.25(0.04)	
G_21-46_	Q6.2	1.4	-0.20(0.04)	
G_21-46_	Q3.2	0.9	-0.15(0.04)	
G_46-120_	Q4.1	4.2	0.33(0.06)	
G_46-120_	Q6.2	2.2		0.50(0.10)
G_120-210_	Q7.1	6.7	-0.32(0.05)	0.21(0.07)
G_120-210_	Q3.1	2.6	0.23(0.05)	
G_46-210_	Q7.1	5.9	-0.33(0.04)	0.19(0.06)
G_46-210_	Q4.1	5.2	0.34(0.04)	
G_46-210_	Q5	2.4	0.23(0.04)	
G_46-210_	Q1.1	1.1	0.16(0.04)	
G_210-240_	Q7.1	2.0	-0.13(0.05)	0.23(0.07)

^i^Percentage residual variance explained by the QTL

^ii^Significant additive (a) and dominance (d) effects. Effects were standardized by dividing by the square root of the residual variance from the base model. Standard errors are given in brackets. A positive additive effect means that the White Duroc allele increased the phenotypic value.

**Fig 2:**
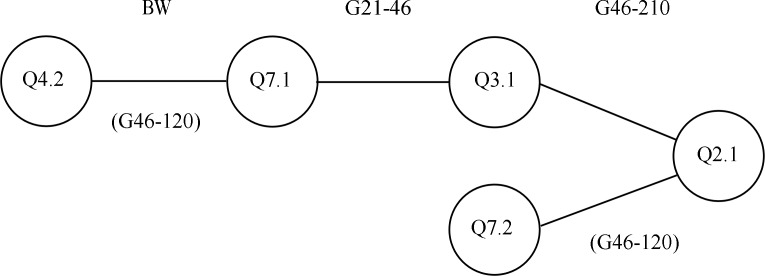
Epistatic network of Q2.1, Q3.1 and Q7.1. Interactions are shown by lines between loci. Ages where epistasis occurred are shown at the top of the diagram. When a pair was pointwise significant at a second age this is shown in brackets underneath the interaction.

Two QTL, Q7.1 and Q3.1, were epistatic at two ages with different loci and a third, Q2.1, interacted with two loci at the same age. Q7.1 interacted with Q4.2 to affect birth weight and was epistatic with Q3.1 for G_21-46_. Q3.1 also interacted with Q2.1 for G_46-210_. Q2.1 was additionally epistatic with Q7.2 in that interval. Taking into account effects at difference ages, these loci formed a small network (Fig 2). The pairs Q7.1 x Q4.2 and Q2.1 x Q7.2 were also pointwise significant for G_46-120_. Q7.1 and Q3.1 had independent effects at other ages. Q7.1 affected G_120-210_, G_46-210_ and G_210-240_. Q3.1 affected G_120-210_.

One QTL, Q4.1, had independent effects at several ages and was epistatic at one age. It affected G_21-46_, G_46-120_ and G_46-210_; and interacted with Q2.2 for G_120-210_. One other QTL, Q6.2, was detected at more than one age. Q6.2 had independent effects on G_21-46_ and G_46-120_.

Four epistatic pairs were pointwise significant when they were tested for effects at other ages. The pairs Q7.1 x Q4.2 and Q2.1 x Q7.2 are described above. Q7.1 x Q4.2 was the only pair with evidence of epistasis at a distinct age from when it was detected. In the other instances, the ages were overlapping. The pair Q1.2 x Q8 was detected for G_120-210_ and was pointwise significant for G_46-210_, and Q13 x Q6.1 was found for G_46-210_ and was pointwise significant for G_120-210_.

**Fig 3:**
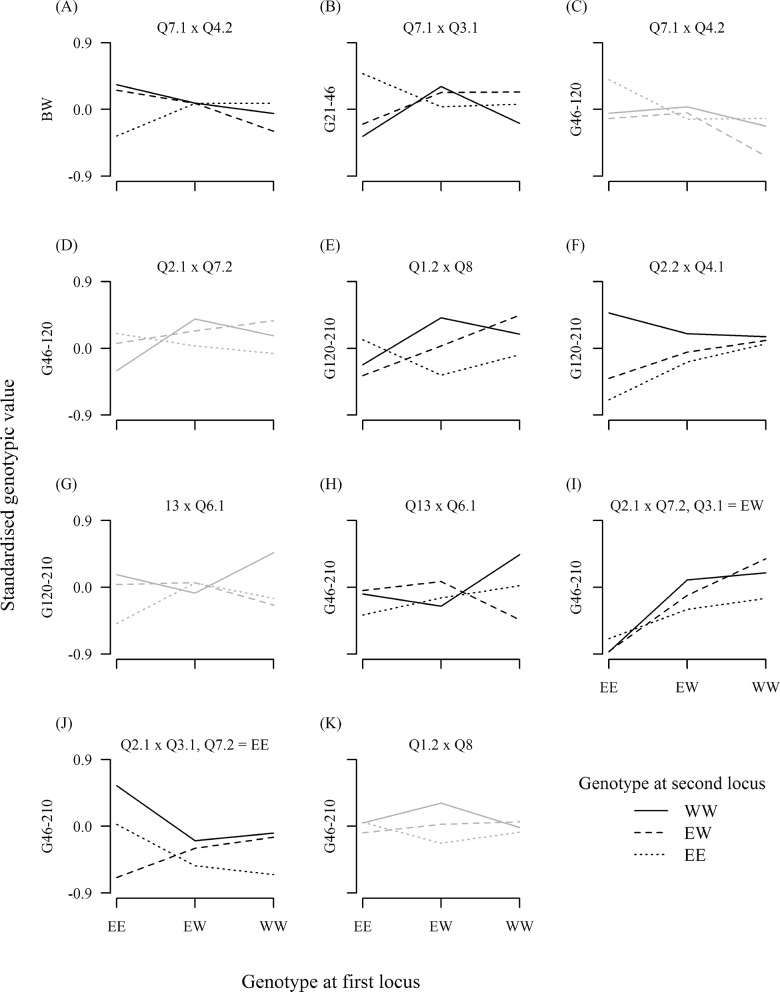
Consequences of epistasis. Standardized genotypic values are shown for the nine genotype combinations at a pair of loci. Values were standardized by dividing them by the square root of the residual variance from the base model. Tick marks on the x-axis give the genotype at the first QTL and different line types represent the genotype at the second QTL. *E* represents the Erhualian allele and *W* the White Duroc allele. Genomewide significant epistasis is shown in black and pointwise significant interactions are shown in grey. The two pairs involving Q2.1 are each illustrated for a selected genotype at the third locus. (A) Epistasis between Q7.1 and Q4.2 for birth weight. (B) Epistasis between Q7.1 and Q3.1 for growth from days 21–46. (C) Epistasis between Q7.1 and Q4.2 for growth from days 46–120. (D) Epistasis between Q2.1 and Q7.2 for growth from days 46–120. (E) Epistasis between Q1.2 and Q8 for growth from days 120–210. (F) Epistasis between Q2.2 and Q4.1 for growth from days 120–210. (G) Epistasis between Q13 and Q6.1 for growth from days 120–210. (H) Epistasis between Q13 and Q6 for growth from days 46–210. (I) Epistasis between Q2.1 and Q7.2 when Q3.1 has genotype *EW* for growth from days 46–210. (J) Epistasis between Q2.1 and Q3.1 when Q7.2 has genotype *EE* for growth from days 46–210. (K) Epistasis between Q1.2 and Q8 for growth from 46–210 days.

Plots of epistasis are shown in Fig 3. We evaluated additive and dominance effects for each locus by genotype at the other locus and describe epistasis by the significant effects (Table 7). Epistasis between Q7.1 and Q4.2 for birth weight switched the direction of the Q7.1 effect. The Erhualian allele at Q7.1 had a positive effect when Q4.2 was heterozygous (*EW*) but a negative effect when Q4.2 was homozygous Erhualian (*EE*, Fig 3A). For Q2.1 interacting with Q7.2 for G_46-210_, Q7.2 changed from having an additive to dominance effect. Q7.2 had a positive effect of the White Duroc allele when Q2.1 was *EW* but a positive dominance effect when Q2.1 was homozygous White Duroc (*WW*, Fig 3I). Pairs Q1.2 x Q8 and Q2.2 x Q4.1, both for G_120-210_, showed some symmetry in effects. For Q1.2 x Q8, the White Duroc allele had positive effects of similar magnitude at both loci when the other locus was *EW* (Fig 3E). For Q2.2 x Q4.1, the White Duroc allele at Q2.2 had a positive effect when Q4.1 was *EE* or *EW* and the White Duroc allele at Q4.1 had a positive effect when Q2.2 was *EE* (Fig 3F). There was one interaction, Q13 x Q6.1 for G_46-210_, where both loci had only dominance effects (Fig 3H).

**Table 7:** Epistatic effects and values estimated in the independent effects model.

Trait	QTL	Interacting QTL	Epistatic effects^i^ (s.e.)	Independent effects^ii^ (s.e.)
BW	Q7.1	Q4.2	**EE**: a = 0.22(0.08), **EW**: a = -0.28(0.06)	a = -0.14(0.04)
BW	Q4.2	Q7.1	**EE**: a = 0.35(0.08)	
G_21-46_	Q7.1	Q3.1	**WW**: d = 0.6(0.2)	a = -0.10(0.04), d = 0.17(0.05)
G_21-46_	Q3.1	Q7.1	**EE**: a = -0.4(0.1)	
G_46-120_	(Q7.1)	(Q4.2)	**EW**: a = -0.25(0.08), d = 0.3(0.1)	a = -0.23(0.05)
G_46-120_	(Q4.2)	(Q7.1)	**WW**: d = -0.5(0.2)	
G_46-120_	(Q2.1)	(Q7.2)	**WW**: d = 0.5(0.2)	
G_46-120_	(Q7.2)	(Q2.1)		
G_120-210_	Q1.2	Q8	**EW**: a = 0.41(0.08)	a = 0.29(0.06)
G_120-210_	Q8	Q1.2	**EW**: a = 0.39(0.08)	
G_120-210_	Q2.2	Q4.1	**EE**: a = 0.4(0.1), **EW**: a = 0.26(0.08)	
G_120-210_	Q4.1	Q2.2	**EE**: a = 0.6(0.1)	a = 0.33(0.05)
G_120-210_	(Q13)	(Q6.1)		
G_120-210_	(Q6.1)	(Q13)		
G_46-210_	Q13	Q6.1	**EW**: d = 0.3(0.1)	
G_46-210_	Q6.1	Q13	**WW**: d = -0.7(0.2)	
G_46-210_	Q2.1	Q7.2, Q3.1	**EWEW**:^iii^ a = 0.6(0.1), **WWEW**: a = 0.5(0.1)	a = 0.22(0.04)
G_46-210_	Q7.2	Q2.1	**EW**: a = 0.20(0.06), **WW**: d = 0.4(0.1)	
G_46-210_	Q3.1	Q2.1	**EE**: d = -1.0(0.3)	a = 0.23(0.05)
G_46-210_	(Q2.1)	(Q8)		
G_46-210_	(Q8)	(Q2.1)	**EW**: a = 0.27(0.06)	a = 0.18(0.04)

Loci that showed pointwise-significant epistasis are indicated by brackets.

^i^Significant additive (a) and dominance (d) epistatic effects. The genotype at the other locus is shown in bold. Effects were standardized by dividing by the square root of the residual variance from the base model. Standard errors are given in brackets. A positive additive effect means that the White Duroc allele increased the phenotypic value.

^ii^For loci that were detected by the independent QTL analysis, significant effects in the independent effects model.

^iii^Two locus genotype, with the first genotype for Q7.2 and the second for Q3.1.

In the genomewide-significant separate epistatic pairs, either both QTL had a significant effect in only one genetic background, or one QTL had an effect in two backgrounds and the other had an effect in one background. In the more complex interaction of Q2.1 with two loci, Q2.1 had similar effects for two Q7.2 genotypes when Q3.1 was heterozygous, Q7.2 had different effects in two backgrounds and Q3.1 had an effect in one background. Additive epistatic effects nearly always occurred in an *EE* or *EW* background. In most cases the White Duroc allele had a positive effect but Q3.1 and Q7.1 showed positive effects of the Erhualian allele. Dominance epistatic effects most often occurred in a *WW* background and nearly always without an additive effect. Both positive and negative dominance epistatic effects were seen.

Independent QTL mostly showed purely additive effects or additive effects with dominance. There was only one case of a dominance effect in the absence of an additive effect. A similar number of independent loci showed positive effects of the Erhualian allele as the White Duroc allele. Q3.2, Q6.2, Q7.1 and Q9 all had beneficial effects of the Erhualian allele.

The effects of Q3.1 and Q7.1 were different in each of their interactions. As described above, in epistasis with Q4.2 for birth weight, Q7.1 had a positive additive effect of the Erhualian allele in an *EW* background and a negative effect in an *EE* background. In epistasis with Q3.1 for G_21-46_, Q7.1 instead had a positive dominance effect in a *WW* background (Fig 3B). In the pointwise-significant epistasis with Q4.2 for G_46-120_, Q7.1 had a positive effect of the Erhualian allele in an *EW* background but unlike the effect for birth weight, the Erhualian allele was also dominant (Fig 3C). When Q7.1 acted independently, it had a positive dominant effect of the Erhualian allele (Table 6) that resembled the effect on G_46-120_ in an *EW* background.

Q3.1 in epistasis with Q7.1 for G_21-46_ had a positive effect of the Erhualian allele in an *EE* background (Fig 3B). For G_46-210_ in epistasis with Q2.1, Q3.1 had a negative dominance effect in an *EE* background (Fig 3J). Acting independently for G_120-210_, Q3.1 had a negative effect of the Erhualian allele, which was in the opposite direction to the epistatic effect on G_21-46_.

Q4.2 interacting with Q7.1 had different effects on birth weight and in the pointwise-significant epistasis for G_46-120_. For birth weight, Q4.2 had a positive additive effect of the White Duroc allele in an *EE* background (Fig 3A). For G_46-120_, Q4.2 had a negative dominance effect in a *WW* background (Fig 3C).

For the other three pairs that were pointwise significant at a second age, the pattern of genotypic values showed similarities between the ages but effects in the original interval were usually not significant in the second and some were much smaller. Q1.2 x Q8 had positive additive effects of both loci in an *EW* background on G_120-210_. For G_46-210_, Q8 had an additive effect but there was little additive effect of Q1.2 (Fig 3E and 3K). Epistasis between Q13 and Q6.1 produced a positive dominance effect of Q13 in an *EW* background and a negative dominance effect of Q6.1 in a *WW* background for G_46-210_. For G_120-210_, neither locus had a significant effect and Q6.1 showed far less dominance (Fig 3G and 3H). The results for Q2.1 x Q7.2 would be expected to differ between G_46-210_ and G_46-120_ because for G_46-210_ Q2.1 also interacted with Q3.1. For G_46-210_, Q2.1 had additive effects when Q7.2 was *EW* or *WW* and Q3.1 was *EW*; Q7.2 had an additive effect in an *EW* background and dominance effect in a *WW* background. For G_46-120_, there was a novel dominance effect of Q2.1 when Q7.2 was *WW*. There were no other significant effects although Q2.1 had a nominally significant (*p* < 0.025) additive effect in a *WW* background and Q7.2 had a nominally significant additive effect in an *EW* background (Fig 3D and 3I).

Q4.1 had similar effects at different ages. When it acted independently, the White Duroc allele additively increased growth (Table 6). In epistasis with Q2.2 for G_120-210_, as described above, Q4.1 had a positive additive effect of the White Duroc allele in an *EE* background (Table 7). Q6.2 had different effects at the two ages where it was detected. For G_21-46_, the Erhualian allele had a positive additive effect. For G_46-120_, Q6.2 had a positive dominance effect (Table 6).

The mean magnitude of significant epistatic effects was 0.39 for additive effects and 0.58 for dominance effects (Table 7). In comparison, the values for significant independent effects were 0.21 and 0.26, respectively (Table 6). Q4.1 and Q2.1 had the largest additive epistatic effects of 0.6 for G_120-210_ and G_46-210_, respectively. Q3.1 had the largest dominance epistatic effect of 1.0 for G_46-210_. For independent QTL, Q4.1 had the largest additive effect of 0.34 for G_46-210_. Q6.2 had the largest independent dominance effect of 0.5 for G_46-120_.

Separate epistatic pairs on average explained 3.5% of the residual phenotypic variance, with the interaction terms explaining 1.5% of the variance (Table 5). The interactions involving Q2.1 explained 5.7% of the residual variance; the two sets of interaction terms explained 1.2% and 0.7% of the variance. Pair Q1.2 x Q8 for G_120-210_ explained the most residual variance (6.8%) and had the highest variance attributable to the interaction terms (2.8%). Independent QTL explained on average 2.8% of the residual phenotypic variance (Table 6). Q7.1 explained the most residual phenotypic variance of an independent QTL at 6.7% for G_120-210_.

In most cases, one locus in an epistatic pair was detected by the independent QTL analysis (Table 7). For the genomewide-significant epistasis of Q13 x Q6.1, neither locus was detected by the independent analysis. There was only one case, Q2.1 x Q3.1 for G_46-210_, where both loci were both found by the independent analysis. In total five QTL were only identified by the epistatic analysis. The epistatic model explained up to 9% more residual variance than a model of the QTL detected by the independent analysis that assumed no interactions (the independent effects model) (Table 8). The proportionally greatest amount of additional residual variance explained was for birth weight, where it was three-quarters the amount explained by the independent effects model.

**Table 8:** Residual variance explained by independent effects model and additional variance explained by epistatic model.

	BW	G_0-21_	G_21-46_	G_46-120_	G_120-210_	G_46-210_	G_210-240_
**Independent**	2.5	1.2	6.9	9.6	17.8	22.5	2.0
**Epistatic**	1.9	-	1.5	4.8	9.0	4.8	-

For epistatic QTL that were detected by the independent QTL analysis, we compared estimated effects between the independent effects and epistatic models. Clearly, an independent effect cannot capture an effect that changes direction under epistasis. In the independent effects model, Q7.1 showed a positive effect of the Erhualian allele on birth weight; the negative effect of this allele was only apparent with epistasis. There were two other effects seen with epistasis but not when a locus was treated as an independent QTL: the dominance effect of Q7.1 for G_46-120_, and dominance of Q3.1 for G_46-210_. When the independent effects model gave an effect of the same type and direction as a significant epistatic effect, the estimated size was on average 0.6 of the epistatic value (Table 7). In two cases, an outcome was observed in the independent effects model that was not significant with epistasis: Q7.1 showed a negative effect of the Erhualian allele on G_21-46_ and Q3.1 had a positive effect of the White Duroc allele on G_46-210_. However, the loci each had a nominally significant epistatic effect in the same direction in one background.

## Discussion

With epistasis included, we investigated QTL for pig growth at a series of ages. The detected QTL explained up to 27% of the residual phenotypic variance. We identified loci on chromosomes 3, 4 and 7 (Q3.1, Q4.1 and Q7.1) that had effects at more than one age. These are likely to be regions that are fundamental to growth because the specific growth processes are expected to change with age. Q3.1, Q7.1 and a locus on chromosome 2 (Q2.1) formed a small network of QTL that were each epistatic with more than one locus. This network is also of interest to explore further.

Q7.1 had effects at the most ages. The only interval where it had no effect was birth to 21 days. Q7.1 was epistatic for growth before 46 days, showed pointwise significant epistasis for 46–120 days and acted independently at the other ages. It explained the most residual phenotypic variance of an independent QTL. Q4.1 affected growth at the second most ages. It influenced growth in all intervals between 21 and 210 days. It predominantly had independent effects but was epistatic for growth from 120–210 days. Q4.1 had the largest independent additive effect. Q3.1 showed effects at three ages. It was epistatic for growth from 21–46 days and 46–210 days and acted independently for 120–210 days. It had the largest dominance effect in epistasis. Q2.1 interacted with two loci in the period of 46–210 days and showed pointwise significant epistasis for 46–120 days. It had the largest additive effect in epistasis.

A locus on chromosome 6 (Q6.2) was the final QTL detected at more than one age, with independent effects in two periods. However the effects were different between these times. The positions identified were 10 cM apart. Therefore, we reason that these are probably two separate loci. We tested all epistatic pairs for pointwise-significant effects at other ages. This indicated one more locus that might have an impact at a second distinct age. It was another position on chromosome 4 (Q4.2) that interacted with Q7.1 for birth weight and was pointwise significant for 46–120 days. However the epistatic effects and the pattern of genotypic values were different at these ages. We conclude that the chromosome 4 locus is probably distinct from the birth weight QTL and the chromosome 7 QTL the same as found at other ages, because the chromosome 7 effects in an *EW* background were similar to the independent effects of Q7.1 at other ages. The resolution from interval mapping is limited, and for any of the loci identified at more than one age, it is possible that they are different genetic elements. The other pairs with pointwise significant effects at a second age were cases where the age intervals overlapped and we feel this is insufficient evidence of effects at more than one age.

As well as identifying a genetic network, evaluating epistasis indicated a more important role for Q2.1 and Q3.1 than was apparent from the independent QTL analysis. The independent analysis detected an effect of Q2.1 on growth from 46–210 days but assessing epistasis showed that this locus interacts with two QTL in that interval. Effects of Q3.1 on growth from 46–210 days and 120–210 days were found in the independent analysis but this might occur because they are overlapping periods. The epistatic analysis revealed that Q3.1 affected growth at a separate age. The number of epistatic QTL is comparable to results for growth in chickens [15,18]. We found more epistasis than was detected for body dimensions and organ weights in this population [8].

We investigated an effect of mother because variations between mothers in milk volume or quality could be important. The mother explained as much as 19% of the phenotypic variance, with the greatest influence on 21–46 and 46–210 days. As this shows, there continued to be an effect of mother after the mothers were removed at 46 days, which suggests the benefits from maternal provision are long lasting. It is also possible that the effect captures differences between families in which QTL alleles are present. Our analysis assumes that the QTL are fixed for alternative alleles in the two breeds but this may not be the case. Some of the pigs were fostered but a full cross fostering experiment would be needed to disentangle maternal resource or environmental effects from genetic effects.

Including mother in the base model had an impact on the results. Without mother, for birth weight, a different QTL on chromosome 7 was detected as epistatic with Q18 and the epistasis between Q7.1 and Q4.2, which was one of the most interesting results, was not found. For growth from 21–46 days, without mother, Q3.2 was not identified and Q7.1 was missed by the independent QTL analysis, although its interaction with Q3.1 was detected. When mother was omitted for growth from days 46–210, the two interactions involving Q2.1 and the effect of Q1.1 were missed and Q7.1 showed epistasis with Q3.2, which we had not detected for this interval. Wei et al. [8] similarly found that including an effect of family could alter the QTL that were detected. For growth from 1–21 days and 210–240 days, omitting mother did not change the QTL that were detected. We did not investigate the effect of removing mother from the models for growth from 46–120 and 120–210 days.

Ai et al. [10] previously analysed the population we investigated for QTL with independent effects on body weight. They reported correlation coefficients between adjacent body weights of 0.59–0.92. QTL may have been detected at more than one age because of these correlations. Loci may also have been identified because of accumulated effects from birth to the measurement time. We instead analysed the increase in body weight between measurements and corrected for effects of start weight. We aimed to specifically detect QTL affecting growth within an age interval. This should allow us to determine the period where a locus influences growth more precisely. We also accounted for more explanatory variables than Ai et al. [10], which as described above for the effect of mother, can identify more loci. For our adjusted phenotypes, excluding G_46-210_ compared to G_46-120_ and G_120-210_, the maximum correlation coefficient between adjacent intervals was only 0.07.

We compared our results with Ai et al.’s [10] findings, excluding QTL that we only identified by our epistatic analysis. The loci they detected for a given age were contrasted with the QTL we found for growth from the previous measurement to that age. QTL they found for day 210 were compared with the loci we identified for either G_46-210_ or G_120-210_. Ai et al. [10] found most QTL for weight at 240 days, where five loci were detected. Three of these, which correspond to Q4.1, Q7.1 and Q8, they also detected for day 210. The other two correspond to our loci Q3.1 and Q5. We found only Q7.1 had a significant effect on growth from 210–240 days but all five loci affected growth from 46–210 or 120–210 days. Hence, we believe most of the effects reported by Ai et al. [10] for body weight at 240 days are caused by correlations between body weights or a cumulative impact of loci over time. In particular, our results indicate that the chromosome 8 QTL they identified at more than one age probably only affected the earlier period.

Ai et al. [10] identified Q7.1 for several ages but reported that its impact was discontinuous, with no significant effect between birth and 210 days. We have shown it has a much broader influence across age. Five QTL that we identified for growth from days 46–210 or 120–210 were not detected by Ai et al. [10] for day 210. Two of these were detected instead for day 240, suggesting they had insufficient power to find them at the previous time point and the power was increased by measuring the cumulative effect over a longer period. The others, including Q2.1, they did not find at all. Ai et al. [10] did not detect one QTL that we found for growth from 21–46 days or an effect of Q6.2 on day 120. They reported one QTL that we did not identify; a locus on chromosome 10 found for day 46.

We propose a new scheme for describing epistasis, where significant additive and dominance effects of a locus in specific backgrounds are given. We think this helps in understanding the outcome of epistasis because it is an extension of the standard representation for single locus effects. Our approach is useful for assessing the consequences of introducing a new allele in a breeding scheme, which depends on the effects of the allele in the genetic backgrounds present. The results show whether the effect of a locus changes direction with the genetic background. Effects at different ages can also be compared, including if the locus acts independently, to evaluate whether they alter with age or the interacting locus.

Previously, epistasis has been described by which of the interaction terms in Eq (2) are significant [19]. For example, epistasis is called dominance by additive if only the dominance by additive coefficient is significant. Dominance by additive epistasis is interpreted as the dominance effect of locus 1 being different when locus 2 is *EE* than *WW*, and the additive effect of locus 2 being different when locus 1 is *EW* rather than *EE* or *WW*. However, Eq (6) shows that the dominance by additive coefficient is not sufficient to determine whether a locus has a significant effect in any of these backgrounds or the direction of the effect. The effect sizes also depend on the values of other coefficients in the model. As an example, the epistasis between Q7.1 and Q3.1 for growth from days 21–46 is dominance by additive. We found that Q7.1 had a positive dominance effect when Q3.1 was *WW* (homozygous White Duroc) but no significant effect when Q3.1 was *EE* (homozygous Erhualian) and Q3.1 had a negative effect of the White Duroc allele when Q7.1 was *EE* but no significant effect in other backgrounds. It is also difficult to understand the overall consequences when more than one interaction coefficient is significant. Two interaction coefficients were significant for 3 of the 7 epistatic pairs. One advantage of defining epistasis by the interaction coefficients is that only four values are tested for significance. For our approach, 12 effects are tested and statistical power is reduced by the multiple-testing correction. However, we found a significant effect for all except one case of epistasis, which was an instance where the interaction was only pointwise significant.

Another way to look at epistasis is by differences in the genotypic values of the nine genotype-combinations [31]. There are 36 pairwise comparisons and hence the multiple-testing penalty is higher than for our approach. We tried this alternative on our genomewide-significant simple pairs. At least one genotype had significantly higher predicted growth than one or more others. But the genotypes could not be split into groups where the phenotypes for one group were significantly different from all the other genotypes. Hence we could not say that some genotypes had significantly higher or lower growth than the rest. For example, for Q1.2 x Q8 affecting growth from 120–210 days, genotype *EWWW* (*EW* at Q1.2 and *WW* at Q8) had higher growth than *EWEE*, *EEEW*, and *WWEE*; and genotype *WWEW* had higher growth than *EWEE* and *EEEW*. None of these had significantly different growth from the four remaining genotypes. The pairwise differences could be complex, as in this example, where the set of differences for several genotypes overlap but do not match. We think this representation is less clear than our scheme, which showed that the White Duroc allele at both Q1.2 and Q8 increased growth when the other locus was heterozygous.

Q7.1 and Q3.1 each had effects in opposite directions. The effect of Q7.1 on birth weight switched direction with the genotype at Q4.2. At other ages, Q7.1 mostly had a positive effect of the Erhualian allele but it also had a nominally significant negative effect of the Erhualian allele on growth from 21–46 days when Q3.1 was heterozygous. This effect was significant in an independent effects model, suggesting that it is a true effect, which we had insufficient power to detect because of our correction for multiple testing. Q3.1 had a positive effect of the Erhualian allele in epistasis with Q7.1 at one age but at another age it acted independently and the Erhualian allele had a negative effect. Q3.1 also had a nominally significant negative effect of the Erhualian allele in a second interaction, which similarly is probably a true effect because it was identified in the independent effects model. These results show that epistasis can substantially modify the effect of a locus and the outcome can be altered by which second locus is involved.

Growth QTL in a similar position to Q7.1 have been detected in crosses of European breeds with Meishan pigs, which are from an area near to the Erhualian [11,13,37,38]. The Meishan allele increased growth in each case. Ai et al. [10] found that the Erhualian allele at this locus increased body weight at several ages. To our knowledge, it is the first time for this locus that an allele from a Chinese breed has been shown to reduce growth. Beeckman et al. [39] detected a QTL for daily gain on chromosome 3 in the region of Q3.1 in a cross of Meishan with the commercial Pietrain breed. They found that the Meishan allele decreased growth.

There are some classical types of epistasis: complementary, duplicate, dominant and recessive [32] that can be explained by simple molecular models [40]. Complementary epistasis exists if the causal allele is needed at both loci to produce an effect. It could be caused by a direct interaction between the protein products of the loci. There is duplicate epistasis when the causal allele at either locus generates an effect. It might occur when loci have similar functions and are able to compensate for each another. Dominant and recessive forms of epistasis resemble Bateson’s original description of an allele at one locus being masked by the effects of an allele at another locus. The effect of the first locus is only seen when the second locus is homozygous for the alternative allele (dominant), or homozygous for the alternative allele or heterozygous (recessive). The definition implies that the effect of the second locus is unaltered by the genotype at the first. It is difficult to see how this could occur for a quantitative trait: if the numerical value of the trait differs between genotypes at the first locus for genotype 1 at the second locus but not genotype 2, changing from genotype 1 to 2 at the second locus must also have a different effect depending on the genotype at the first. Or considered in terms of the epistasis plots, if one line is horizontal and the other slopes (effect of first locus), the vertical distance between the lines (effect of second locus) must change.

The interaction between Q2.2 and Q4.1 is duplicate epistasis. The other pairs did not match any of the classical types. None of the different pairs of loci had the same set of significant effects or a similar pattern of genotype values. The genomewide significant pairs usually had a significant effect of one locus in one or two backgrounds and significant effect of the other locus in one background. Unlike in the classical forms, there were dominance effects without additive effects and additive effects in a heterozygous background but not in a homozygous background. Except for Q2.2 x Q4.1, we are not able to suggest biological mechanisms that could explain the epistasis results. Cordell [41] advised that statistical detection of epistasis is unlikely to be informative about the nature of any underlying molecular interactions. The outcomes we found illustrate this problem. It seems that to gain biological insight from epistasis found in QTL mapping, extensive follow-up work will be needed to identify the genes and understand their function.

Three epistatic pairs were pointwise significant in a second interval that overlapped with the period where they were detected. These did not have the same effects at the two ages. In two cases, effects found in the original interval were greatly reduced in the second. We believe this is because of the timescale over which epistasis occurs relative to the measured intervals. We investigated shorter intervals of 46–120 days and 120–210 days and a longer interval of 46–210 days. If epistasis happens over a long period but is measured in a shorter interval, there will be less time for the consequences to build up and some effects may not be seen. If instead, epistasis acts in a small interval but is measured over a longer one, the overall impact will be lessened and some effects may become negligible. There was also one epistatic pair and two independent QTL detected for growth from 46–210 days that were not significant in either of the shorter periods; and an epistatic pair and independent QTL, each seen in one of the short periods, were not significant in the longer interval. Our results show that different QTL and effects can be identified when other measurement intervals are used. Therefore, the ages when measurements are taken should be based on periods of biological or economic interest. Q2.1 interacting with Q7.2 had an effect in the second period that was not significant for the original interval. This appears to be because Q2.1 was also epistatic with Q3.1 in the original interval and the power was reduced by fitting additional interaction terms.

We evaluated the relative importance of epistasis for growth in this population in several ways. Firstly, we compared the overall residual phenotypic variance explained by epistatic and independent loci. Epistatic loci explained more of the residual variance than independent QTL at three ages. The maximum residual variance explained by epistatic QTL was 17% for growth from 120–210 days, compared to 9% explained by independent loci. Secondly, we looked at the additional residual variance explained by the model with epistasis over a model based on the results from the independent QTL analysis. This will be a lower value because some of the epistatic loci were detected by the independent analysis. The highest additional variance explained by the epistatic model was 9%. Thirdly, we measured the residual variance explained by separate epistatic pairs and each independent QTL. The pairs explained a quarter more residual variance on average than the independent QTL. Finally, we contrasted the size of significant effects between epistatic and independent loci. Epistatic effects were on average about twice the size of the effects of independent QTL.

Testing all pairs of loci for epistasis is computationally demanding and the power to find interactions is substantially reduced by correcting for multiple testing. Hence, some studies have only tested for interactions between loci already detected by an independent QTL analysis [42,43]. Such an approach is appealing because the number of loci examined is likely to rise as SNP chips and next generation sequencing are utilised. In human genetics, genomewide association studies (GWAS) that test hundreds of thousands of markers will probably be expanded to consider epistasis. A GWAS strategy where markers are first identified in a single locus analysis, although with a more liberal than usual significance threshold, and only interactions between these loci are assessed has been proposed [44]. The expectation appears to be that epistatic loci influencing complex human diseases will show individual effects [45]. We found one epistatic pair where neither locus was detected by an independent QTL analysis. Each locus had a dominance effect in one background. The amount of residual variance explained by the interaction of these QTL was the highest of the pairs found at that age, and the third highest of all pairs. There was only one instance where both QTL in a pair were found by the independent analysis and the interaction between these loci explained the lowest residual variance of the genomewide-significant pairs. Most pairs comprised a locus identified by the independent analysis and a novel locus. Five loci were only found by the epistatic analysis. Our results show that restricting tests of epistasis to loci with measurable individual effects can miss influential QTL.

As some epistatic loci can be found by an independent QTL analysis, we examined the extent to which effects could be misinterpreted if epistasis was not investigated. The most serious outcome would be an effect appearing to be in the opposite direction. This happened with Q7.1 for birth weight, which showed a positive effect of the Erhualian allele in an independent effects model but was epistatic with Q4.2 and had a negative effect of the Erhualian allele when Q4.2 was homozygous Erhualian. Two other loci had effects that were not apparent in the independent model. Another error is assuming that a locus has an effect when it is negligible in some backgrounds. For most of our loci, there was a large effect in one background and the effect was much smaller in other backgrounds. Finally, the size of effects can be underestimated because an independent effects model measures averages across genetic backgrounds. We found that significant effects from the independent model were on average 0.6 of the size of significant epistatic effects. It is important that QTL effects are accurately estimated when loci are being considered for breeding programmes. Errors in estimates could result in programmes failing because of unexpected consequences, minimal improvement, or in beneficial QTL being overlooked.

## Conclusions

We have identified QTL on chromosomes 2, 3, 4 and 7 that are likely to have important roles in growth. Epistasis was sufficient to switch the direction of the chromosome 7 locus effect on birth weight and to cause the chromosome 3 QTL to have effects in opposite directions. These consequences are biologically interesting as well as pertinent to breeding schemes and should be investigated further including exploring the molecular mechanisms. Generally, epistasis produced a significant effect of one locus in one or two backgrounds and a significant effect of the other locus in one background. The outcomes were different for each pair and age and only one pair showed a classical type of epistasis. Epistatic QTL made a considerable contribution to the residual variance explained and had larger effects on average than independent loci. Many of the epistatic loci were not detected by a standard QTL scan. For those that were identified, failing to account for epistasis underestimated significant effects, missed effects and did not show that effects were absent in some backgrounds. Our results motivate greater inclusion of epistasis in QTL studies.

## Supporting Information

S1 Text Additional equations. Equations for the genotypic values and effects of a QTL interacting with two other loci. Equations for the standard errors of estimated epistatic effects.
(DOCX)

S2 Text *A* and *D* values. Tab delimited text file. Columns are labelled in the format chromosome.position(in cM).A or chromosome.position.D.
(TXT)

S3 Text Birth weight and explanatory variables. Tab delimited text file.
(TXT)

S4 Text Growth from birth to 21 days and explanatory variables. Tab delimited text file.
(TXT)

S5 Text Growth from 21 to 46 days and explanatory variables. Tab delimited text file.
(TXT)

S6 Text Growth from 46 to 120 days and explanatory variables. Tab delimited text file.
(TXT)

S7 Text Growth from 120 to 210 days and explanatory variables. Tab delimited text file.
(TXT)

S8 Text Growth from 46 to 210 days and explanatory variables. Tab delimited text file.
(TXT)

S9 Text Growth from 210 to 240 days and explanatory variables. Tab delimited text file.
(TXT)

S1 Table QTL positions on original Haldane map. (DOCX)
