# Supplementary material for: Consequences of Epistasis on Growth in an Erhualian × White Duroc Pig Cross
Source: PLoS One. 2017 Jan 6;12(1):e0162045. doi: 10.1371/journal.pone.0162045 (PMC5218402; doi:10.1371/journal.pone.0162045)
Supplement: S1 Text — Equations for the genotypic values and effects of a QTL interacting with two other loci. Equations for the standard errors of estimated epistatic effects. (DOCX) [file pone.0162045.s001.docx]

### Genotypic values for a QTL interacting with two loci

For locus 1 interacting with both loci 2 and 3, the genotypic values are given by

(1)

where, for example, is the value for genotype EE at the first locus, WW at the second locus and EW at the third locus, is the additive by additive interaction coefficient for epistasis between loci 1 and 2 and is the additive (at locus 1) by dominance (at locus 3) interaction coefficient for epistasis between loci 1 and 3.

### Epistatic effects for a QTL interacting with two loci

The epistatic effects and , of locus 1, for genotype at the second locus and genotype at the third locus are given by

### (2)

### Standard errors of epistatic additive and dominance values

The standard error of an estimated value is the square root of the variance of the estimate. The variance of a variable constructed from a linear combination of other variables has the property

, (3)

where is the variance of variable, is the covariance of variables and and is a constant. The variances for the epistatic additive and dominance values for a pair of loci are given by

(4)

For locus 1 interacting with two other loci, the variances are given by


 (5)
