# Supplementary material for: Consequences of Epistasis on Growth in an Erhualian × White Duroc Pig Cross
Source: PLoS One. 2017 Jan 6;12(1):e0162045. doi: 10.1371/journal.pone.0162045 (PMC5218402; doi:10.1371/journal.pone.0162045)
Supplement: S1 Table — (DOCX) [file pone.0162045.s010.docx]

| **QTL** | **Chromosome** | **Position, cM** | **Trait** |
| --- | --- | --- | --- |
| **Q1.1** | 1 | 54 | G_46-210_ |
| **Q1.2** | 1 | 131 | G_120-210_ |
|  |  | (131) | (G_46-210_) |
| **Q2.1** | 2 | (13) | (G_46-120_) |
|  |  | 13 | G_46-210_ |
| **Q2.2** | 2 | 64 | G_120-210_ |
| **Q3.1** | 3 | 71 | G_21-46_ |
|  |  | 63 | G_46-210_ |
|  |  | 93 | G_120-210_ |
| **Q3.2** | 3 | 145 | G_21-46_ |
| **Q4.1** | 4 | 87 | G_21-46_ |
|  |  | 86 | G_46-120_ |
|  |  | 79 | G_120-210_ |
|  |  | 78 | G_46-210_ |
| **Q4.2** | 4 | 161 | BW |
|  |  | (161) | (G_46-120_) |
| **Q5** | 5 | 132 | G_46-210_ |
| **Q6.1** | 6 | (40) | (G_120-210_) |
|  |  | 40 | G_46-210_ |
| **Q6.2** | 6 | 131 | G_21-46_ |
|  |  | 141 | G_46-120_ |
| **Q7.1** | 7 | 61 | BW |
|  |  | 67 | G_21-46_ |
|  |  | 61 | G_46-120_ |
|  |  | 66 | G_120-210_ |
|  |  | 60 | G_46-210_ |
|  |  | 66 | G_210-240_ |
| **Q7.2** | 7 | (169) | (G_46-120_) |
|  |  | 169 | G_46-210_ |
| **Q8** | 8 | 58 | G_120-210_ |
|  |  | 58 | G_46-210_ |
| **Q9** | 9 | 92 | G_0-21_ |
| **Q13** | 13 | (75) | (G_120-210_) |
|  |  | 75 | G_46-210_ |
| **Q18** | 18 | 24 | BW |

Brackets indicate QTL that showed pointwise-significant epistasis and were not detected by the independent QTL analysis.
